# Comparative biochemical analysis of recombinant reverse transcriptase enzymes of HIV-1 subtype B and subtype C

**DOI:** 10.1186/1742-4690-7-80

**Published:** 2010-10-07

**Authors:** Hong-Tao Xu, Yudong Quan, Eugene Asahchop, Maureen Oliveira, Daniella Moisi, Mark A Wainberg

**Affiliations:** 1 McGill University AIDS Centre, Lady Davis Institute for Medical Research, Jewish General Hospital, Montreal, Quebec, Canada; 2 Departmens of Medicine, McGill University, Montreal, Quebec, Canada; 3 Department of Microbiology and Immunology, McGill University, Montreal, Quebec, Canada

## Abstract

**Background:**

HIV-1 subtype C infections account for over half of global HIV infections, yet the vast focus of HIV-1 research has been on subtype B viruses which represent less than 12% of the global pandemic. Since HIV-1 reverse transcriptase (RT) is a major target of antiviral therapy, and since differential drug resistance pathways have been observed among different HIV subtypes, it is important to study and compare the enzymatic activities of HIV-1 RT derived from each of subtypes B and C as well as to determine the susceptibilities of these enzymes to various RT inhibitors in biochemical assays.

**Methods:**

Recombinant subtype B and C HIV-1 RTs in heterodimeric form were purified from *Escherichia coli *and enzyme activities were compared in cell-free assays. The efficiency of (-) ssDNA synthesis was measured using gel-based assays with HIV-1 PBS RNA template and tRNA_3_^Lys ^as primer. Processivity was assayed under single-cycle conditions using both homopolymeric and heteropolymeric RNA templates. Intrinsic RNase H activity was compared using 5'-end labeled RNA template annealed to 3'-end recessed DNA primer in a time course study in the presence and absence of a heparin trap. A mis-incorporation assay was used to assess the fidelity of the two RT enzymes. Drug susceptibility assays were performed both in cell-free assays using recombinant enzymes and in cell culture phenotyping assays.

**Results:**

The comparative biochemical analyses of recombinant subtype B and subtype C HIV-1 reverse transcriptase indicate that the two enzymes are very similar biochemically in efficiency of tRNA-primed (-) ssDNA synthesis, processivity, fidelity and RNase H activity, and that both enzymes show similar susceptibilities to commonly used NRTIs and NNRTIs. Cell culture phenotyping assays confirmed these results.

**Conclusions:**

Overall enzyme activity and drug susceptibility of HIV-1 subtype C RT are comparable to those of subtype B RT. The use of RT inhibitors (RTIs) against these two HIV-1 enzymes should have comparable effects.

## Introduction

Human immunodeficiency virus type 1 (HIV-1) genetic diversity is reflected by the existence of three groups (M, N, and O), of which group M is responsible for greater than 90% of HIV-1 infections. Currently, there are at least nine group M subtypes (A, B, C, D, F, G, H, J, and K) and numerous recombinant forms that show 25-35% overall genetic variation that includes 10-15% variability in reverse transcriptase (RT) [1,2]. Subtype C variants of HIV-1 are responsible for over 50% of the worldwide pandemic, and largely represent the dominant viral species in Sub-Saharan Africa and India [3]. Despite this, no work has yet been reported on the comparative biochemistry of RT enzymes derived from either subtype B or C. Most data have been inferred from enzymatic studies on prototypic subtype B viruses [4].

HIV-1 RT is a multi-functional enzyme that possesses both RNA- and DNA-directed DNA polymerase activities as well as an RNase H activity [5]. Due to its key role in HIV-1 replication, RT has been a major target for development of antiviral drugs. RT inhibitors (RTIs) are core constituents of antiretroviral (ARV) regimens and include both nucleoside and nucleotide RTIs (NRTIs), the first of which was zidovudine (ZDV) [6]. Currently, eight NRTIs and four non-nucleoside reverse transcriptase inhibitor (NNRTIs) are approved for treatment of HIV-1 infection. The former are activated by host enzymes to their active triphosphate forms (diphosphate for tenofovir), which bind to the active site of RT, acting as competitive inhibitors of RT and interfering with the addition of incoming nucleosides to growing viral DNA chains. The NNRTIs are non-competitive inhibitors that bind allosterically to an asymmetric and hydrophobic cavity, about 10 Å away from the catalytic site of the HIV-1 RT [7]. RNase H is responsible for degradation of the RNA template after the synthesis of minus-strand strong stop (-ss) DNA [8] and is also a potential target for drug discovery [9]. Despite remarkable progress in the development of antivirals, the occurrence of drug resistance remains a problem in the management of HIV infection.

RT exists as a heterodimer that consists of 66 kDa (p66) and 51 kDa (p51) subunits. The p51 subunit shares the same N-terminal sequence, as does p66, but lacks the C-terminal 140 amino acids of the latter. Although p51 provides RT with essential structural and conformational stability, p66 is the catalytically active subunit and includes the N-terminal polymerase domain (residues 1-321) and C-terminal RNase H domain (residues 441-560), linked by a connection domain (cn) (residues 322-440) [7]. All of these domains can be involved in drug resistance [10]. Enzymatic studies using purified subtype B recombinant RT have provided valuable information on catalytic properties and mechanisms of resistance [11].

Differences among subtypes can occur in the development of and interactions among drug resistance mutations. This genetic diversity has the potential to influence rates of development of drug resistance and relevant mutational pathways [12-15]. Although antiretroviral drugs have been designed based on subtype B RT, this is the first report of a comparative biochemical analysis of the subtype B and C RT enzymes.

## Results

### Purification of recombinant HIV-1 RTs from subtype B and subtype C

**Figure 1:**
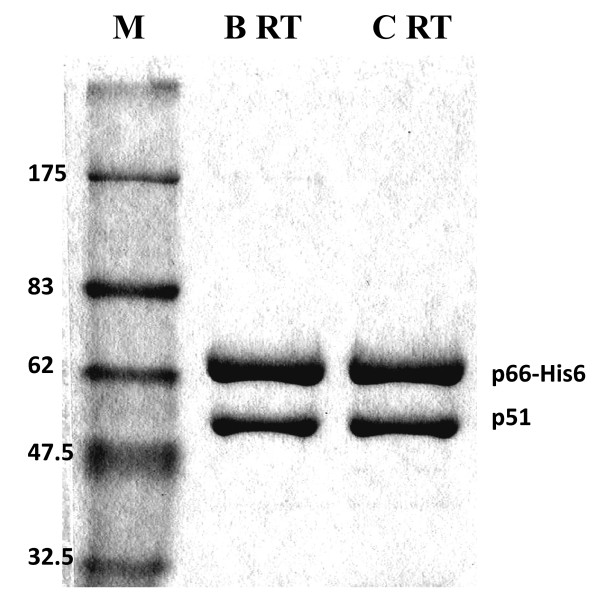
**Purified recombinant heterodimer RT enzymes of subtypes B and C were analyzed by 8% SDS-PAGE after Coomassie-Brilliant Blue staining**. M (molecular weight markers in kilodaltons are shown on the left); B RT/C RT, (subtype B/C HIV-1 wild-type RTs). The positions of purified recombinant RT heterodimer subunits of both subtypes that possessed a similar ratio of p51/p66 are indicated on the right.

The subtype C HIV-1 RT sequence used in this study differs from consensus subtype B RT by 6.96% of amino acids. Thirty-nine amino acids were variable in subtype C RT, of which 16 were in the DNA polymerase domain (residues 1-321), 12 were in the connection domain (residues 322-440) and 11 were in the RNase H domain (residues 441-560). This level of variation is in agreement with previous reports showing that HIV-1 RT subtypes differ from one another by ≈ 5%-6% of amino acids [16]. By co-expression of the HIV-1 protease (PR) with the RT coding sequence in one plasmid and through use of the well-established method of immobilized metal affinity chromatography (IMAC), followed by ion-exchange chromatography [17], recombinant heterodimeric (p66/p51) RTs of both subtypes B and C were purified to >95% homogeneity and shown to possess similar molar ratios (Figure 1.). This indicates that the amino acid polymorphisms in subtype C RT do not affect protease cleavage, p66/p51 heterodimer formation, or RT purification [18]. Through individual expression of the p66 and p51 subunits from separate plasmids and mixing the *E. coli *cell paste containing both subunits prior to cell lysis, various labs have obtained homogenous HIV-1 RT heterodimers [19-21]. This strategy has also been used for purification of heterodimeric HIV-1 RTs from different subtypes [22]. The results presented here show that the one plasmid co-expression strategy is effective, non-laborious, and convenient, especially for the simultaneous biochemical analysis of a large panel of RTs of different subtypes. The inclusion of a 6-His tag in recombinant RT enzymes has been shown to be devoid of deleterious effects on polymerase, RNase H, tRNA binding, and RT inhibitor susceptibilities [23-25].

To determine the specific activity of the recombinant enzyme preparations, DNA polymerase activity was measured using synthetic poly(rA)/oligo(dT)_12-18 _template/primer with variable amounts of RT enzymes over a time-course reaction. The calculated initial velocities were then divided by the concentration of enzyme used in the assay to determine the specific activity of the recombinant RT preparations. Recombinant RTs from HIV-1 subtype B and subtype C shared similar activities at 238 units/μg and 233 units/μg respectively. This result also confirmed the efficiency of the expression and purification procedure.

### Susceptibilities of HIV-1 subtype B and subtype C recombinant RTs and viruses to RT inhibitors

To test whether NRTIs and NNRTIs have similar inhibitory effects on HIV-1 subtype B and C RT, recombinant RT heterodimers were analyzed in cell-free RNA-dependent DNA polymerase assays in the presence of NRTIs ZDV-TP, 3TC-TP, and TFV-DP, or NNRTIs NVP, EFV, and ETR. The results of Table 1 show that both the subtype B and the two subtype C RT enzymes from isolates BG05 and M01 (GenBank accession number AF492609 and AF492603) were inhibited to similar extent by all of the RT inhibitors tested. The reason for using two RTs of subtype C was to reduce the possibility of natural variability. We also performed phenotypic assays in cord blood mononuclear cells using wild-type viruses from both subtypes and found that they shared similar susceptibilities to all of the RT inhibitors tested i.e. ZDV, 3TC,TFV, NVP, EFV and ETR (Table 2), in agreement with previous data [26].

**Table 1:** RT inhibitor susceptibilities for HIV-1 subtype B and subtype C recombinant RTs

RT inhibitors	**IC**_**50**_** (μM)**^**a**^			
	
	**B RT-1**^**b**^	**B RT-2**^**c**^	**C RT-1 **^**d**^	**C RT-2 **^**e**^
ZDV-TP	3.1 ± 0.5	2.9 ± 0.4	4.3 ± 0.4	3.8 ± 0.5

3TC-TP	2.7 ± 0.3	4.2 ± 0.5	3.7 ± 0.6	4.1 ± 0.4

TFV-DP	2.5 ± 0.3	2.1 ± 0.3	2.6 ± 0.5	2.9 ± 0.4

NVP	3.2 ± 0.4	5.3 ± 1.4	3.3 ± 0.4	4.3 ± 0.3

EFV	0.11 ± 0.03	0.22 ± 0.03	0.20 ± 0.02	0.18 ± 0.02

ETR	0.17 ± 0.03	0.16 ± 0.02	0.15 ± 0.04	0.19 ± 0.03

^a ^Data represent means ± standard deviations of three determinations.

^b ^NL4-3

^c ^HXB2

^d ^Isolate BG05 (GenBank accession number AF492609)

^e ^Isolate M01 (GenBank accession number AF492603)

**Table 2:** RT inhibitor susceptibilities for HIV-1 subtype B and subtype C viruses

RT inhibitors	**EC**_**50 **_**(nM)**^**a**^	
	
	Subtype B HIV-1	Subtype C HIV-1
ZDV	12.9 ± 5.9	34.8 ± 12.9

3TC	45.4 ± 16.4	105.9 ± 40.2

TFV	334.8 ± 140.9	304.8 ± 93.1

NVP	50.2 ± 29.6	170.4 ± 79.5

EFV	0.6 ± 0.2	1.3 ± 0.4

ETR	0.9 ± 0.4	0.5 ± 0.01

^a ^Data represent means ± standard deviations of three determinations.

### Efficiency of (-) ssDNA synthesis from the natural tRNA_3_^Lys ^primer

**Figure 2:**
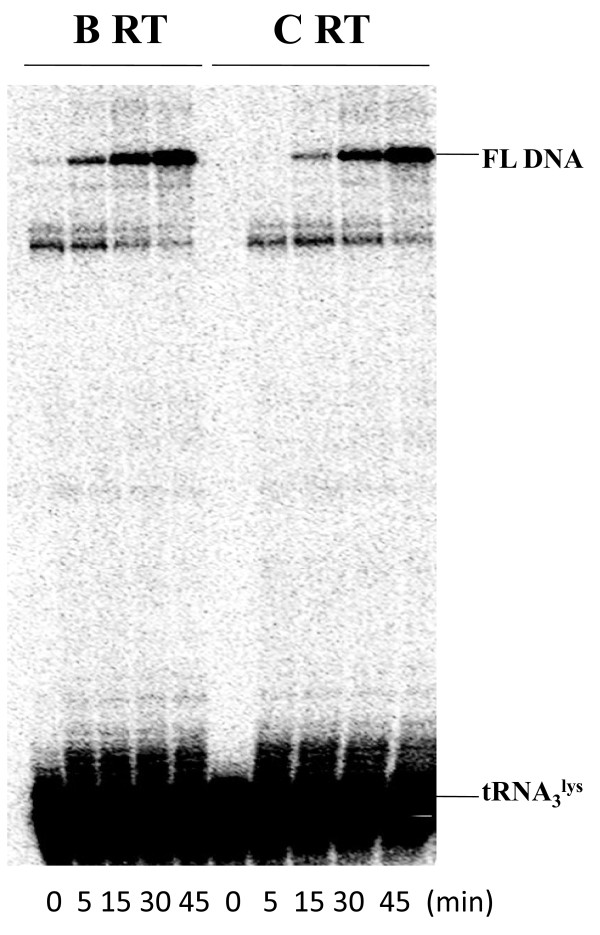
**Efficiency of tRNA**_**3**_^**Lys **^**-primed (-) ssDNA synthesis in cell-free assays**. The efficiencies of synthesis of (-) ssDNA with HIV-1 subtype B and subtype C wild-type RTs were compared in time course experiments using 5'-end ^32^P-labeled human natural tRNA_3_^Lys ^as primer and HIV-1 PBS RNA as template. The HIV-1 PBS RNA template used in this system consists of 258 nucleotides (nt) at the 5' end of the HIV-1 genome, which contains the R, U5, and PBS regions. Synthesis of full-length DNA (FL DNA) by recombinant RT enzymes was monitored in time-course experiments. Reactions were initiated by the addition of MgCl_2 _and dNTPs and stopped at different time points during a period of 45 min. The position of the full-length DNA product (FL DNA) is shown on the right.

The first step in reverse transcription requires human tRNA_3_^Lys ^as primer, which is annealed to a region near the 5'-end of viral RNA termed the primer binding site (PBS). The efficiency of tRNA-primed synthesis of minus-strand strong stop (-) ssDNA correlates with viral replication competence, and this step can sometimes be impeded by the presence of drug resistance mutations [27,28]. We therefore investigated whether the two RT enzymes exhibited differences in the efficiency of (-) ssDNA synthesis by using a HIV-1 PBS RNA template and 5'-end ^32^P labeled tRNA_3_^Lys ^primer. Full-length DNA products were monitored in time course reactions. Figure 2 shows that both enzymes displayed similar levels of tRNA-primed synthesis of (-) ssDNA. This result also indicates that the two enzymes exhibited similar efficiency in regard to tRNA-primed (-) ssDNA synthesis.

### Processivity analysis of recombinant HIV-1 subtype B and subtype C RTs

**Figure 3:**
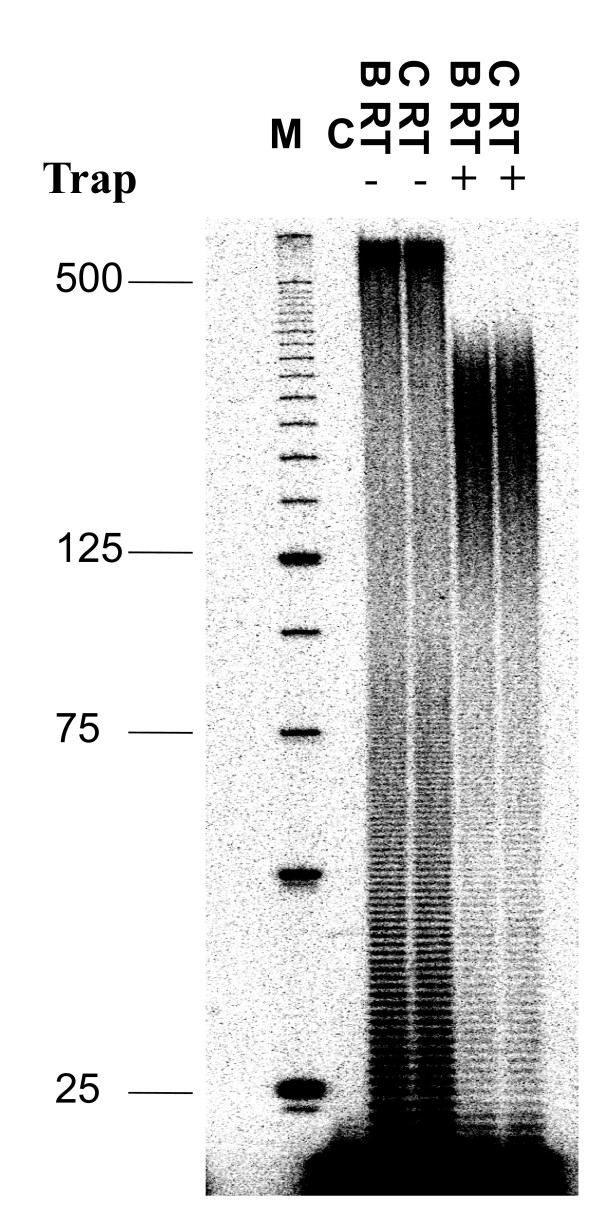
**Processivity assay using a homopolymeric RNA template**. Processivity of the recombinant HIV-1 subtype B and C RT enzymes was assessed using homopolymeric RNA template poly (rA) and oligo (dT)_12-18 _DNA primer. The DNA primer was labeled with ^32^P at the 5'-terminus and annealed to poly (rA) RNA template at an equimolar ratio. Processivities were analyzed by monitoring the size distribution of DNA products in fixed-time experiments in the presence of heparin trap. Parallel reactions were run in the absence of trap to ensure that similar amounts of enzyme activities were present in the reactions. The sizes of some fragments of the standard are indicated on the left side of the panel. All reaction products were resolved by denaturing 6% polyacrylamide gel electrophoresis and visualized by phosphorimaging. M: molecular size standards. C: control reaction in which the heparin trap was preincubated with substrates before the addition of RT.

**Figure 4:**
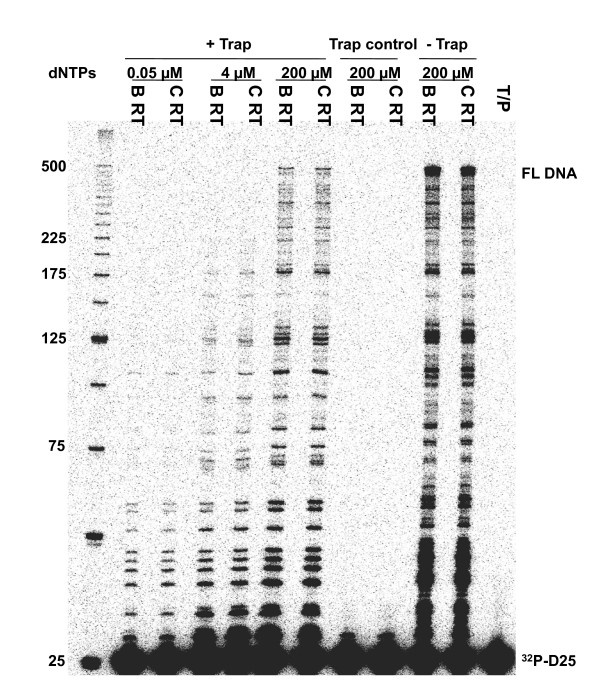
**Processivity assay using a heteropolymeric HIV PBS RNA template**. Processivity of the recombinant HIV-1 subtype B and C RT enzymes was assessed using heteropolymeric HIV PBS RNA template and D25 DNA primer. The DNA primer D25 was labeled with ^32^P at the 5'-terminus and annealed to the RNA template at an equimolar ratio. Processivities were analyzed by monitoring the size distribution of DNA products in fixed-time experiments at three different concentrations of dNTPs in the presence of heparin trap (+ Trap). Parallel reactions were run in the absence of trap (- Trap) at 200 μM of dNTPs to ensure that similar amounts of enzyme activities were present in the reactions. The sizes of some fragments of the ^32^P-labeled 25bp DNA ladder (Invitrogen) in nucleotide (nt) bases are indicated on the left side of the panel. All reaction products were resolved by denaturing 6% polyacrylamide gel electrophoresis and visualized by phosphorimaging. Trap control: control reactions in which the heparin trap was preincubated with substrates before the addition of RT enzymes. T/P: control reaction in which no RT enzymes were included. Positions of ^32^P -labeled D25 primer (^32^P -D25) and the 471-nt full-length extension DNA (FL DNA) product are indicated on the right.

The processivity of a polymerase is defined as the number of nucleotides incorporated in a single round of binding, elongation, and dissociation. Earlier studies showed that HIV replication efficiency is related in part to RT processivity [29,30]. We compared the enzyme processivity of the two subtype RT enzymes by using homopolymeric poly (rA) RNA template (average length 500 nt) annealed to 5' ^32^P-labeled oligo dT primers in a fixed-time experiment in the presence of heparin trap to ensure that each synthesized DNA molecule resulted from a single processive cycle. Figure 3 shows that both enzymes share similar processivity on the homopolymeric RNA template within a size range of the longest products at 160 nt-260 nt. We also compared the processivity of the two RT enzymes using a heteropolymeric RNA template under three different concentrations of dNTPs. The results of Figure 4 clearly demonstrate that both enzymes possessed similar processivity at all three dNTP concentrations dNTPs tested. Primary subtype C HIV-1 isolates have been reported to be less fit than subtype B isolates in PBMCs, CD4+ T cells, and macrophages [31], and these differences seem to be related to lesser efficiency at host cell entry [32]. The results presented here show that subtype C RT does not have a processivity defect compared to subtype B RT.

### Misincorporation efficiency by HIV-1 subtype B and subtype C RTs

**Figure 5:**
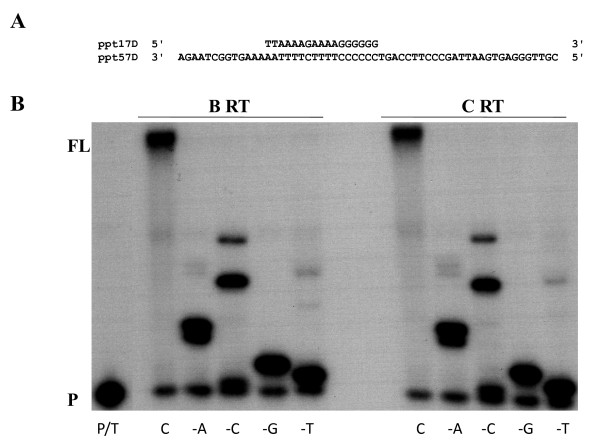
**Misincorporation assay**. (A) Graphic representation of the template and primer system used to monitor the misincorporation efficiency of recombinant subtype B and C RT enzymes. The ^32^P-labeled 17-mer primer ppt17D annealed to 57-mer DNA template ppt57D was extended by HIV-1 subtype B and C recombinant RTs at 37°C for 5 min. The extension reactions were performed in the presence of all four complementary dNTPs, or, alternatively, in the absence of one of the dNTPs. The lanes marked with -A, -G, -C and -T indicate the missing nucleotide. Lanes marked with C indicate that all four dNTPs were included in the dNTP mix. Both RTs displayed similar levels of primer extension in the presence of all four dNTPs. P and FL indicate the positions of unextended primer and full-length extended products, respectively.

HIV-1 RT has low fidelity compared with RTs of other retroviruses and cellular DNA polymerases. Point mutations in HIV-1 RT may strongly affect the fidelity of HIV-1 RT, and we and others have shown that the fidelity of DNA polymerization of M184V-mutated HIV-1 RT is significantly higher than that of wild-type RT [33]. In order to assess the fidelity of recombinant subtype B and C RTs, we performed misincorporation experiments to monitor primer extension in the absence of a single dNTP complementary to various template nucleotides. This misincorporation assay employs a primer extension protocol that qualitatively monitors both misinsertion and mismatch extension in the presence of biased dNTP pools containing only three of the four natural dNTPs. Under these conditions, the elongation of the primer past a template nucleotide complementary to the excluded dNTP requires the insertion of an incorrect nucleotide (misincorporation) and further extension of the generated mismatch primer (mispair extension). In the absence of one of the dNTPs, primer extension stalls one base before the template nucleotide for which the complementary dNTP is withheld (stop site). A higher efficiency of primer extension beyond the stop site reflects a higher ability to utilize incorrect dNTPs, i.e. lower fidelity of the RT. When incubated with mixtures of only three dNTPs in the presence of template-primer ppt57D/ppt17D, both subtype C and B RTs catalyzed substantial extension past the stop sites on the template, indicating low fidelity (Figure 5). However, under conditions that excluded one complementary dNTP, both RTs catalyzed similar extensions beyond the stop. In particular, both RTs showed the highest levels of extension in the minus-dCTP reaction, followed by those involving minus-dATP, minus-dTTP and minus-dGTP. These results show that subtype B and C RT possess a similar degree of fidelity.

### RNase H activity

**Figure 6:**
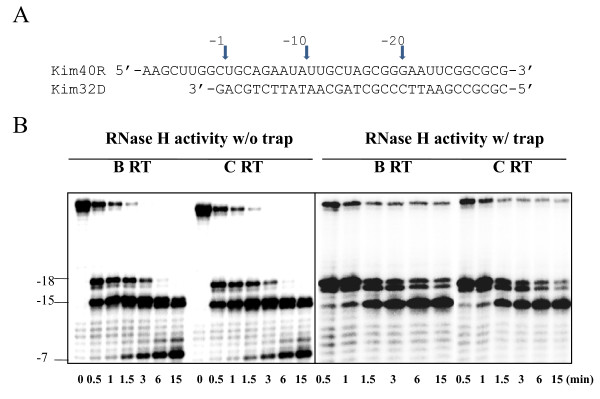
**RNase H activity of HIV-1 subtype B and C recombinant wild type RTs**. (A) Graphic representation of the substrate RNA/DNA (kim40R/kim32D) duplex used to monitor the RNase H cleavage efficiency of both recombinant RTs. The 40-mer RNA kim40R was labeled at its 5'-terminus by ^32^P and annealed to 32-mer DNA oligo kim32D. -1, -10 and -20 are used as markers to indicate the positions of cleavage sites relative to the 3' end of the DNA primer. (B) The RNA-DNA substrate was incubated with the recombinant subtype B and C RT enzymes in assay buffer as described in Materials and Methods. RNase H cleavage was initiated by the addition of MgCl_2 _and analyzed by monitoring substrate cleavage in time-course experiments in the absence (left panel) or presence (right panel) of a heparin trap. The position of cleaved products is indicated on the left. All reactions were resolved by denaturing 6% polyacrylamide gel electrophoresis.

RNase H activity is an integral part of RT function and is essential for viral replication [34]. Mutations abrogating the degradation of template RNA can impact resistance levels against certain NRTIs [35]. Therefore, we compared the intrinsic RNase H activities of subtype B and C RTs using a 40-mer RNA template that was ^32^P-labeled at its 5'-end and annealed to an unlabeled 32-mer DNA primer, such that there was a 8-nt overhang at the 5'-end of the RNA. Equivalent amounts of RT activity were added to the template primer and incubated in the absence of dNTPs. Time-course experiments were employed to compare RNase H cleavage efficiencies in the context of the two RT enzymes. Figure 6 shows that both RTs displayed similar patterns and rates of template cleavage, indicating that they share a common profile of RNase H activity.

## Discussion

This manuscript represents the first attempt of its type to directly compare RT enzymes of different subtypes in regard to processivity, fidelity, RNase H activity, and susceptibility to RT inhibitors. Moreover, our analysis has been conducted using both homopolymeric and heteropolymeric templates. We have further documented that few differences exist among the various RT enzymes studied in regard to each of these characteristics.

These findings are important because of the possibility that factors that relate to polymorphisms within RT could potentially be responsible for appearance of mutations related to drug resistance and/or susceptibilities to HIV inhibitors in a manner that would distinguish between HIV subtypes. Were such differences to be important in regard to enzyme processivity and/or other characteristics of biochemical behaviour, it might follow, in turn, that different therapeutic regimens might be recommended for different HIV subtypes. The fact that few such differences exist suggests that the same anti-RT drugs used to treat subtype B infections should have equal relevance to HIV infections of other subtypes. This relieves a major concern and is of clinical significance. On the other hand, differences in regard to viral template sequences can directly lead to differential appearance of resistance mutations [14,36,37]. This notwithstanding, choices of antiretroviral therapies to be used in therapy should not be affected. Of course, relevant considerations in such decision-making include drug efficacy and tolerability as well as convenience of dosing.

The fact that different mutations may sometimes appear differentially in regard to viruses of different subtypes may have implications in regard to secondary treatment strategies in the aftermath of treatment failure. This is a different topic than that of the initial use of antiretroviral drugs discussed here, and may also have implications for transmitted drug resistance. This reinforces the need to conduct genotyping prior to commencement of antiretroviral therapy in newly infected individuals and/or individuals about to undergo therapy for the first time.

The fact that RT polymorphisms do not appear to impact on enzyme function, as studied by multiple methods in this manuscript, is encouraging news in regard to future development of antiretroviral drugs. Previous findings from our laboratory have also indicated a paucity of differences among HIV integrase enzymes of different subtypes in regard to both 3'-processing and strand-transfer activities [38]. Future studies should be carried out to document that polymorphisms have little or no effect on the behaviour of HIV-1 and other retroviral proteases, but such work has yet to be carried out. It is important, however, to note that previous studies have suggested that resistance to HIV-1 protease inhibitors can occur along different mutational pathways as a function of HIV-1 subtype [39]. The current manuscript allays concerns that functional biochemical differences in RT might play an important role in regard to antiretroviral drug susceptibility.

## Conclusion

Our results provide biochemical evidence that RT enzymes from HIV-1 subtypes B and C share similar catalytic activities in regard to each of (-) ssDNA synthesis, processivity of DNA polymerization, efficiency of misincorporation, and RNase H activity. RT enzymes and viruses from both subtypes were inhibited by NRTIs and NNRTIs to a similar extent. These findings are supportive of the use of recombinant RTs of either subtype for enzyme analysis, drug design, and for studying mechanisms of drug resistance.

## Materials and methods

### Chemicals, cells and nucleic acids

Poly(rA)/oligo(dT)_12-18, _oligo dT_12-18, _ultrapure dNTPs and NTPs were purchased from GE Healthcare. [^3^H]-dTTP (70-80 Ci/mmol) and [γ-^32^P]-ATP were from Perkin Elmer Life Sciences. Natural human tRNA_3_^Lys ^purified from placenta by high-pressure liquid chromatography (HPLC) was purchased from BIO S&T (Montreal, Quebec, Canada). A HIV-1 PBS RNA template spanning the 5' UTR to the primer binding site (PBS) was *in vitro *transcribed from BSSH II-linearized pHIV-PBS DNA [40] by using a T7-MEGAshortscript kit (Ambion, Austin, TX) as described [41].

The oligonucleotides used in this study were synthesized by Integrated DNA Technologies Inc and purified by 6% polyacrylamide-7M urea gel electrophoresis and the sequences are as follows:

D25, 5'-GGATTAACTGCGAATCGTTCTAGCT-3';

dPR, 5'-GTCCCTGTTCGGGCGCCA-3';

ppt17D, 5'-TTAAAAGAAAAGGGGGG-3';

pp57D, 5'-CGTTGGGAGTGAATTAGCCCTTCCAGTCCCCCCTTTTCTTTTAAAAAGTGGCTAAGA-3';

kim40R, 5'-AAGCTTGGCTGCAGAATATTGCTAGCGGGAATTCGGCGCG-3';

kim32D, 5'-CGCGCCGAATTCCCGCTAGCAATATTCTGCAG-3';

Tenofovir (TFV) and tenofovir diphosphate (TFV-DP) were kindly provided by Gilead Sciences (Foster City, California, USA). Zidovudine (ZDV), lamivudine (3TC), ZDV-TP, and 3TC-TP were gifts of Glaxo-SmithKline Inc. Etravirine (ETR) was a gift of Tibotec Inc. Efaverenz (EFV) and nevirapine (NVP) were obtained from Bristol-Myers Squibb Inc. and Boehringer Ingelheim Inc, respectively.

### Recombinant reverse transcriptase expression and purification

The plasmid pRT6H-PROT [17] of which the RT coding region is from HIV-1 HXB2 was kindly provided by Dr. S. F. J. Le Grice. For construction of subtype C and subtype B HIV-1 using RT heterodimer expression plasmids pcRT6H-PROT and pbRT6H-PROT, the RT coding regions of subtype C HIV-1 isolate BG05 (GenBank accession number AF492609) or subtype B HIV-1 pNL4-3 (GenBank accession number AF324493) were subcloned into pRT6H-PROT by standard PCR cloning procedure to replace the original RT coding region [41]. The accuracy of the RT coding sequence was verified by DNA sequencing. Another subtype C RT preparation from isolate M01 (GenBank accession number AF492603) was prepared as reported previously [42]. Recombinant RTs were expressed and purified as described with minor modifications [17,23]. In brief, RT expression in *E. coli *M15 (pREP4) (Qiagen, Mississauga, ON) was induced with 1 mM isopropyl-*b*-D-thiogalactopyranoside (IPTG) at room temperature. The pelleted bacteria were lysed under native conditions with BugBuster Protein Extraction Reagent containing benzonase (Novagen, Madison, WI) according to the manufacturer's instructions. After clarification by high speed centrifugation, the clear supernatant was subjected to the batch method of Ni-NTA metal-affinity chromatography (QIA*expressionist*) (Qiagen). All buffers contained Complete protease inhibitor cocktail (Roche). Histidine-tagged RT was eluted with an imidazole gradient. RT-containing fractions were pooled, passed through DEAE-Sepharose (GE Healthcare), and further purified using SP-Sepharose (GE Healthcare, Mississauga, ON). Fractions containing purified RT were pooled, dialyzed against storage buffer (50 mM Tris-HCl (pH 7.8], 50 mM NaCl and 50% glycerol), and concentrated to 4 mg-8 mg/ml with Centricon Plus-20 MWCO30 kDa (Millipore). Aliquots of proteins were stored at -80°C. Protein concentration was measured by a Bradford protein assay kit (Bio-Rad Laboratories) and the purity of the recombinant RT preparations was verified by SDS-PAGE.

### Specific activity determination

The polymerase activity of each recombinant RT preparation was evaluated in duplicate as described [42] using varying amounts of RTs and a synthetic homopolymeric poly (rA)/p (dT)_12-18 _template/primer (Midland Certified Reagent Company). Each 50-μl reaction contained 0.5 U/ml poly(rA)/p(dT)_12-18_, 50 mM Tris-HCl pH 7.8, 60 mM KCl, and 6 mM MgCl_2_. Reactions were initiated by adding 5 μM dTTP with 5 μCi [^3^H]-dTTP (70-80 Ci/mmol, Perkin Elmer). Aliquots of 15 μl were removed at 3, 7 and 15 min to ensure linearity of the reaction and quenched by the addition of ice-cold 10% trichloroacetic acid containing 20 mM sodium pyrophosphate. After 30-min incubation on ice, aliquots were filtered using 1.2-μm glass fiber type C filter multi-well plates (Millipore) and washed sequentially with cold 10% trichloroacetic acid and ethanol. The extent of radionucleotide incorporation was then determined by liquid scintillation spectrometry. The amount of incorporated [^3^H]-dTTP was plotted as cpm versus time and specific activities were determined from the slopes of the linear regression analyses. An active unit of RT was defined as the amount of enzyme that incorporates 1 pmol of dTTP in 10 min at 37°C.

### RT inhibitor susceptibility assays

Susceptibility to both NRTI and NNRTI inhibitors was assayed using recombinant RT enzymes and heterodimeric HIV-1 PBS RNA template/dPR primer system as described previously [42]. Briefly, RT reaction buffer containing 50 mM Tris-HCl (pH 7.8), 6 mM MgCl_2_, 60 mM KC1, dNTPs (5 μM each) with 2.5 μCi of [^3^H]-dTTP (70-80 mCi/mmol), 30 nM heterogeneous HIV-1 RNA template/primer, 10 units of RT, and variable amounts of RT inhibitors was included in 50-μl reaction volumes. In each reaction, 0, 0.1, 0.3, 1.0, 3.0, 10.0, 30.0 and 100.0 μM of RT inhibitors were added for ZDV-TP, 3TC-TP, TFV-DP and NVP while 0, 0.01, 0.03, 0.10, 0.30, 1.00,3.00 and 10.00 μM were added for EFV and ETR. The reactions were incubated at 37°C for 30 min and the reactions were terminated by adding 0.2 ml of 10% cold trichloracetic acid (TCA) and 20 mM sodium pyrophosphate and incubated for at least 30 min on ice. The precipitated products were filtered through a 96-well MutiScreen HTS FC filter plate (Millipore) and sequentially washed with 200 μl of 10% TCA and 150 μl of 95% ethanol. The radioactivity of incorporated products was analyzed by liquid scintillation spectrometry using a 1450 MicroBeta TriLux Microplate Scintillation and Luminescence Counter (Perkin Elmer). The 50% inhibitory concentration (IC_50_) of each RTI was determined by nonlinear regression analysis using GraphPad Prism software. For determination of RT sensitivities to ZDV-TP, 150 μM sodium pyrophosphate was included in each reaction.

### Phenotypic RT inhibitor susceptibility assays

Phenotypic analysis of RT inhibitor susceptibility was performed with wild type HIV-1 subtype B and Subtype C viruses in a cell-based in vitro assay. Briefly, cord blood mononuclear cells were infected for 2 h with various viral isolates and plated in 96-well plates, at a density of 5 × 106 cells per well, in the presence of each RT inhibitor. The drug concentration ranges for the inhibitors tested were as follows: ZDV (6.4-400 nM), 3TC (3.2-2000 nM), TFV (16-10000 nM), NVP (3.2-2000 nM), EFV (0.05-160 nM), ETR (0.05-160 nM). After 3 days in culture, the culture wells were refreshed with media containing the corresponding drug dilutions. After 7 days, the culture supernatants were collected and analyzed for RT activity to determine the dose response curve. The EC_50 _(50% drug effective concentration) was calculated using GraphPad Prism software [12].

### Efficiency of (-) ssDNA synthesis primed by tRNA_3_^Lys^

Using a cell-free system, the efficiencies of synthesis of (-) ssDNA by HIV-1 subtype B and subtype C RT enzymes were monitored using human natural tRNA_3_^Lys ^(Bio S&T, Lachine, Quebec, Canada) and an HIV-1 PBS RNA primer-template system [40]. The PBS RNA was *in vitro *transcribed from BSSH II-linearized pHIV-PBS DNA by using T7-Megashortscript kit (Ambion, Austin, TX) as described [41]. Human tRNA_3_^Lys^, purified by HPLC from placenta, was labeled at its 5'-end with [γ-^32^P]-ATP using a KinaseMax kit (Ambion) according to the manufacturer's instructions and heat annealed to the RNA template by incubation for 2 min at 95°C followed by 10 min at 70°C and slowly cooling to room temperature as described [28], with the modification that a 30 μl mixture was used that contained 50 mM Tris-HCl (pH 7.8), 50 mM NaCl, 50 nM tRNA_3_^Lys^, 50 nM ^32^P-labeled template PBS RNA and RT enzymes. Synthesis of (-) ssDNA was initiated by the addition of 6 mM MgCl_2 _and dNTPs. Aliquots were removed at different time points and the reactions were stopped by adding 4 volumes of formamide sample buffer (96% of formamide, 0.05% each of bromophenol blue and xylene cyanol FF and 20 mM EDTA). The products were separated on 6% polyacrylamide-7 M urea gels and were exposed to x-ray film after gel drying. The intensity of gel bands was analyzed with Scion Image software (Scion Corp., Frederick, MD).

### Processivity assays

The processivity of recombinant RT proteins was analysed using both homopolymeric and heteropolymeric RNA templates in the presence of a heparin enzyme trap to ensure a single processive cycle, i.e., a single round of binding and of primer extension and dissociation. Assays on homopolymeric RNA were performed as described elsewhere [29,43]. The primer-templates were annealed by heating the solution of ^32^P-end-labeled oligo dT_12-18 _(GE Healthcare) with an equimolar concentration of poly (rA) homopolymeric RNA template (GE Healthcare) to 90°C for 2 min and incubating the solution for an additional 10 min at 70°C, followed by slow cooling to room temperature. RT enzymes and T/Ps were preincubated for 5 min at 37°C in the same buffer system as described above for (-)ssDNA synthesis. Reactions were initiated by the addition of dTTP and heparin trap (final concentration 2 mg/ml) and incubated at 37°C for 10 min; 2 μl of reaction mixture were removed and mixed with 8 μl of formamide sample buffer (90% formamide, 10 mM EDTA, and 0.1% each of xylene cyanol and bromophenol blue). Reaction products were heat denatured and analyzed by 6% denaturing polyacrylamide gel electrophoresis and phosphorimaging. The effectiveness of the trap was assessed and verified in pilot experiments in which the heparin trap at various concentrations was preincubated with substrates before the addition of RT enzymes.

In assays performed on heteropolymeric RNA, HIV RNA template was prepared in vitro using the MEGAscript™ transcription kit (Ambion, Austin, TX) from ACC I-linearized plasmid pHIV-PBS DNA, which consists of a 497-base pair HIV-1 sequence spanning the R region of the HIV-1 long terminal repeat and a portion of the gag region [40]. The 25-nt DNA primer D25 is complementary to the 5' end of the gag sequence. The primers were [γ-^32^P]-ATP-labeled and filtered by NucAway spin column (Ambion, Austin, TX). The template/primer complex was prepared as follows: the template and primer were mixed at a molar ratio of 1:1, denatured at 85°C for 5 min, and then sequentially cooled to 55°C for 8 min and 37°C for 5 min to allow for specific annealing of primer to the template. Reactions were performed as above except that three different concentrations of dNTPs were used.

### Misincorporation assay

The template-primer ppt57D/ppt17D was used to determine the extent of misincorporation in the absence of one complementary dNTP. The 17-mer DNA primer ppt17D was ^32^P-labeled at the 5'end by [γ-^32^P]-ATP using a KinaseMax Kit (Ambion) and annealed to the 57-mer DNA template at a molar ratio of 1:3. Reaction mixtures (20 μl) contained 50 nM template/primer, recombinant RT enzymes at equal activities, 50 mM Tris·HCl, pH 7.8, 60 mM KCl, and 6 mM MgCl_2_. Reactions were incubated at 37°C for 5 min in the presence of all four dNTPs (250 μM each) or in the presence of 3 dNTPs by excluding one complementary dNTP. Reactions were stopped by adding 4 volumes of formamide sample buffer (96% of formamide, 0.05% each of bromophenol blue and xylene cyanol FF and 20 mM EDTA). The products were denatured by heating at 90°C for 3 min, separated on 6% polyacrylamide-7 M urea gels, and exposed to x-ray film after gel drying.

### RT-catalyzed RNase H Activity

Intrinsic RNase H assays were performed as reported [44]. RNase H activity was assayed on 40-mer 5'-end ^32^P-labeled heteropolymeric RNA template kim40R annealed to the complementary 32-mer DNA oligomer kim32D at a 1:4 molar ratio [45]. Reactions were conducted at 37°C in mixtures containing 200 nM RNA-DNA duplex substrate with equal RT activities in assay buffer of 50 mM Tris-HCl, pH 7.8, 60 mM KCl, in the presence or absence of heparin trap (2 mg/ml). Reactions were initiated by adding 1/10 vol of 50 mM MgCl_2. _Aliquots were removed at different times after initiation of reactions and quenched by adding 4 volumes of formamide loading dye. The samples were heated at 90°C for 3 min, cooled on ice, and electrophoresed through 6% polyacrylamide-7M urea gels. The gels were analyzed by phosphorimaging. The efficacy of the heparin trap was verified by pre-incubation experiments performed by 10-min preincubation of various concentrations of heparin trap with substrates in the presence of magnesium followed by initiation of the reaction with RT enzymes.

## Competing interests

The authors declare that they have no competing interests.

## Authors' contributions

MAW supervised the project and corrected the manuscript. HX and YQ purified the enzymes, performed biochemical experiments, and drafted the manuscript. EA and MO performed phenotypic analyses. DM performed sequencing reactions. All authors read and approved the final manuscript.

## References

[B1] Rambaut A Posada D Crandall KA Holmes EC The causes and consequences of HIV evolution Nat Rev Genet 2004 5 52 61 10.1038/nrg1246 14708016

[B2] Robertson DL Anderson JP Bradac JA Carr JK Foley B Funkhouser RK Gao F Hahn BH Kalish ML Kuiken C HIV-1 nomenclature proposal Science 2000 288 55 56 10.1126/science.288.5463.55d 10766634

[B3] Esparza J Bhamarapravati N Accelerating the development and future availability of HIV-1 vaccines: why, when, where, and how? Lancet 2000 355 2061 2066 10.1016/S0140-6736(00)02360-6 10885368

[B4] Hemelaar J Gouws E Ghys PD Osmanov S Global and regional distribution of HIV-1 genetic subtypes and recombinants in 2004 AIDS 2006 20 W13 23 10.1097/01.aids.0000247564.73009.bc 17053344

[B5] Goff SP Retroviral reverse transcriptase: synthesis, structure, and function J Acquir Immune Defic Syndr 1990 3 817 831 1694894

[B6] Fischl MA Richman DD Grieco MH Gottlieb MS Volberding PA Laskin OL Leedom JM Groopman JE Mildvan D Schooley RT The efficacy of azidothymidine (AZT) in the treatment of patients with AIDS and AIDS-related complex. A double-blind, placebo-controlled trial N Engl J Med 1987 317 185 191 10.1056/NEJM198707233170401 3299089

[B7] Kohlstaedt LA Wang J Friedman JM Rice PA Steitz TA Crystal structure at 3.5 A resolution of HIV-1 reverse transcriptase complexed with an inhibitor Science 1992 256 1783 1790 10.1126/science.1377403 1377403

[B8] Schultz SJ Champoux JJ RNase H activity: structure, specificity, and function in reverse transcription Virus Res 2008 134 86 103 10.1016/j.virusres.2007.12.007 18261820 PMC2464458

[B9] Tramontano E Esposito F Badas R Di Santo R Costi R La Colla P 6-[1-(4-Fluorophenyl)methyl-1H-pyrrol-2-yl)]-2,4-dioxo-5-hexenoic acid ethyl ester a novel diketo acid derivative which selectively inhibits the HIV-1 viral replication in cell culture and the ribonuclease H activity in vitro Antiviral Res 2005 65 117 124 10.1016/j.antiviral.2004.11.002 15708638

[B10] Johnson VA Brun-Vezinet F Clotet B Gunthard HF Kuritzkes DR Pillay D Schapiro JM Richman DD Update of the drug resistance mutations in HIV-1: December 2009 Top HIV Med 2009 17 138 145 20068260

[B11] Sarafianos SG Marchand B Das K Himmel DM Parniak MA Hughes SH Arnold E Structure and function of HIV-1 reverse transcriptase: molecular mechanisms of polymerization and inhibition J Mol Biol 2009 385 693 713 10.1016/j.jmb.2008.10.071 19022262 PMC2881421

[B12] Brenner BG Oliveira M Doualla-Bell F Moisi DD Ntemgwa M Frankel F Essex M Wainberg MA HIV-1 subtype C viruses rapidly develop K65R resistance to tenofovir in cell culture AIDS 2006 20 F9 13 10.1097/01.aids.0000232228.88511.0b 16816549

[B13] Gupta RK Chrystie IL O'Shea S Mullen JE Kulasegaram R Tong CY K65R and Y181C are less prevalent in HAART-experienced HIV-1 subtype A patients AIDS 2005 19 1916 1919 10.1097/01.aids.0000189860.36688.e5 16227803

[B14] Invernizzi CF Coutsinos D Oliveira M Moisi D Brenner BG Wainberg MA Signature nucleotide polymorphisms at positions 64 and 65 in reverse transcriptase favor the selection of the K65R resistance mutation in HIV-1 subtype C J Infect Dis 2009 200 1202 1206 10.1086/605894 19764886

[B15] Martinez-Cajas JL Pant-Pai N Klein MB Wainberg MA Role of genetic diversity amongst HIV-1 non-B subtypes in drug resistance: a systematic review of virologic and biochemical evidence AIDS Rev 2008 10 212 223 19092977

[B16] Gonzales MJ Machekano RN Shafer RW Human immunodeficiency virus type 1 reverse-transcriptase and protease subtypes: classification, amino acid mutation patterns, and prevalence in a northern California clinic-based population J Infect Dis 2001 184 998 1006 10.1086/323601 11574914 PMC2597357

[B17] Le Grice SF Gruninger-Leitch F Rapid purification of homodimer and heterodimer HIV-1 reverse transcriptase by metal chelate affinity chromatography Eur J Biochem 1990 187 307 314 10.1111/j.1432-1033.1990.tb15306.x 1688798

[B18] Stahlhut MW Olsen DB Expression and purification of retroviral HIV-1 reverse transcriptase Methods Enzymol 1996 275 122 133 full_text 9026635 10.1016/s0076-6879(96)75010-3

[B19] Maier G Dietrich U Panhans B Schroder B Rubsamen-Waigmann H Cellai L Hermann T Heumann H Mixed reconstitution of mutated subunits of HIV-1 reverse transcriptase coexpressed in Escherichia coli - two tags tie it up Eur J Biochem 1999 261 10 18 10.1046/j.1432-1327.1999.00304.x 10103027

[B20] Pandey VN Kaushik N Rege N Sarafianos SG Yadav PN Modak MJ Role of methionine 184 of human immunodeficiency virus type-1 reverse transcriptase in the polymerase function and fidelity of DNA synthesis Biochemistry 1996 35 2168 2179 10.1021/bi9516642 8652558

[B21] Stahlhut M Li Y Condra JH Fu J Gotlib L Graham DJ Olsen DB Purification and characterization of HIV-1 reverse transcriptase having a 1:1 ratio of p66 and p51 subunits Protein Expr Purif 1994 5 614 621 10.1006/prep.1994.1084 7532052

[B22] Held DM Kissel JD Thacker SJ Michalowski D Saran D Ji J Hardy RW Rossi JJ Burke DH Cross-clade inhibition of recombinant human immunodeficiency virus type 1 (HIV-1), HIV-2, and simian immunodeficiency virus SIVcpz reverse transcriptases by RNA pseudoknot aptamers J Virol 2007 81 5375 5384 10.1128/JVI.01923-06 17329328 PMC1900219

[B23] Le Grice SF Cameron CE Benkovic SJ Purification and characterization of human immunodeficiency virus type 1 reverse transcriptase Methods Enzymol 1995 262 130 144 full_text 8594344 10.1016/0076-6879(95)62015-x

[B24] Kim B Hathaway TR Loeb LA Fidelity of mutant HIV-1 reverse transcriptases: interaction with the single-stranded template influences the accuracy of DNA synthesis Biochemistry 1998 37 5831 5839 10.1021/bi972672g 9558316

[B25] Kim B Ayran JC Sagar SG Adman ET Fuller SM Tran NH Horrigan J New human immunodeficiency virus, type 1 reverse transcriptase (HIV-1 RT) mutants with increased fidelity of DNA synthesis. Accuracy, template binding, and processivity J Biol Chem 1999 274 27666 27673 10.1074/jbc.274.39.27666 10488107

[B26] Fleury HJ Toni T Lan NT Hung PV Deshpande A Recordon-Pinson P Boucher S Lazaro E Jauvin V Lavignolle-Aurillac V Susceptibility to antiretroviral drugs of CRF01_AE, CRF02_AG, and subtype C viruses from untreated patients of Africa and Asia: comparative genotypic and phenotypic data AIDS Res Hum Retroviruses 2006 22 357 366 10.1089/aid.2006.22.357 16623640

[B27] Diallo K Marchand B Wei X Cellai L Gotte M Wainberg MA Diminished RNA primer usage associated with the L74V and M184V mutations in the reverse transcriptase of human immunodeficiency virus type 1 provides a possible mechanism for diminished viral replication capacity J Virol 2003 77 8621 8632 10.1128/JVI.77.16.8621-8632.2003 12885880 PMC167213

[B28] Wei X Liang C Gotte M Wainberg MA The M184V mutation in HIV-1 reverse transcriptase reduces the restoration of wild-type replication by attenuated viruses AIDS 2002 16 2391 2398 10.1097/00002030-200212060-00003 12461412

[B29] Back NK Nijhuis M Keulen W Boucher CA Oude Essink BO van Kuilenburg AB van Gennip AH Berkhout B Reduced replication of 3TC-resistant HIV-1 variants in primary cells due to a processivity defect of the reverse transcriptase enzyme EMBO J 1996 15 4040 4049 8670908 PMC452124

[B30] Caliendo AM Savara A An D DeVore K Kaplan JC D'Aquila RT Effects of zidovudine-selected human immunodeficiency virus type 1 reverse transcriptase amino acid substitutions on processive DNA synthesis and viral replication J Virol 1996 70 2146 2153 8642636 10.1128/jvi.70.4.2146-2153.1996 PMC190052

[B31] Ball SC Abraha A Collins KR Marozsan AJ Baird H Quinones-Mateu ME Penn-Nicholson A Murray M Richard N Lobritz M Comparing the ex vivo fitness of CCR5-tropic human immunodeficiency virus type 1 isolates of subtypes B and C J Virol 2003 77 1021 1038 10.1128/JVI.77.2.1021-1038.2003 12502818 PMC140829

[B32] Marozsan AJ Moore DM Lobritz MA Fraundorf E Abraha A Reeves JD Arts EJ Differences in the fitness of two diverse wild-type human immunodeficiency virus type 1 isolates are related to the efficiency of cell binding and entry J Virol 2005 79 7121 7134 10.1128/JVI.79.11.7121-7134.2005 15890952 PMC1112120

[B33] Diallo K Gotte M Wainberg MA Molecular impact of the M184V mutation in human immunodeficiency virus type 1 reverse transcriptase Antimicrob Agents Chemother 2003 47 3377 3383 10.1128/AAC.47.11.3377-3383.2003 14576091 PMC253767

[B34] Wendeler M Lee HF Bermingham A Miller JT Chertov O Bona MK Baichoo NS Ehteshami M Beutler J O'Keefe BR Vinylogous ureas as a novel class of inhibitors of reverse transcriptase-associated ribonuclease H activity ACS Chem Biol 2008 3 635 644 10.1021/cb8001039 18831589 PMC2941776

[B35] Ehteshami M Gotte M Effects of mutations in the connection and RNase H domains of HIV-1 reverse transcriptase on drug susceptibility AIDS Rev 2008 10 224 235 19092978

[B36] Coutsinos D Invernizzi CF Xu H Moisi D Oliveira M Brenner BG Wainberg MA Template usage is responsible for the preferential acquisition of the K65R reverse transcriptase mutation in subtype C variants of human immunodeficiency virus type 1 J Virol 2009 83 2029 2033 10.1128/JVI.01349-08 19073730 PMC2643749

[B37] Harrigan PR Sheen CW Gill VS Wynhoven B Hudson E Lima VD Lecocq P Aguirre R Poon AF Sluis-Cremer N Silent mutations are selected in HIV-1 reverse transcriptase and affect enzymatic efficiency AIDS 2008 22 2501 2508 10.1097/QAD.0b013e328318f16c 19005273 PMC4829083

[B38] Bar-Magen T Donahue DA McDonough EI Kuhl BD Faltenbacher VH Xu H Michaud V Sloan RD Wainberg MA HIV-1 subtype B and C integrase enzymes exhibit differential patterns of resistance to integrase inhibitors in biochemical assays AIDS 2010 in press 10.1097/QAD.0b013e32833cf265 20647908

[B39] Lisovsky I Schader SM Martinez-Cajas JL Oliveira M Moisi D Wainberg MA HIV-1 protease codon 36 polymorphisms and differential development of resistance to nelfinavir, lopinavir, and atazanavir in different HIV-1 subtypes Antimicrob Agents Chemother 2010 54 2878 2885 10.1128/AAC.01828-09 20404123 PMC2897293

[B40] Arts EJ Li X Gu Z Kleiman L Parniak MA Wainberg MA Comparison of deoxyoligonucleotide and tRNA(Lys-3) as primers in an endogenous human immunodeficiency virus-1 in vitro reverse transcription/template-switching reaction J Biol Chem 1994 269 14672 14680 7514178

[B41] Xu HT Martinez-Cajas JL Ntemgwa ML Coutsinos D Frankel FA Brenner BG Wainberg MA Effects of the K65R and K65R/M184V reverse transcriptase mutations in subtype C HIV on enzyme function and drug resistance Retrovirology 2009 6 14 19210791 10.1186/1742-4690-6-14 PMC2644664

[B42] Quan Y Brenner BG Marlink RG Essex M Kurimura T Wainberg MA Drug resistance profiles of recombinant reverse transcriptases from human immunodeficiency virus type 1 subtypes A/E, B, and C AIDS Res Hum Retroviruses 2003 19 743 753 10.1089/088922203769232548 14585205

[B43] Sharma PL Crumpacker CS Decreased processivity of human immunodeficiency virus type 1 reverse transcriptase (RT) containing didanosine-selected mutation Leu74Val: a comparative analysis of RT variants Leu74Val and lamivudine-selected Met184Val J Virol 1999 73 8448 8456 10482597 10.1128/jvi.73.10.8448-8456.1999 PMC112864

[B44] Gopalakrishnan V Peliska JA Benkovic SJ Human immunodeficiency virus type 1 reverse transcriptase: spatial and temporal relationship between the polymerase and RNase H activities Proc Natl Acad Sci USA 1992 89 10763 10767 10.1073/pnas.89.22.10763 1279694 PMC50422

[B45] Operario DJ Balakrishnan M Bambara RA Kim B Reduced dNTP interaction of human immunodeficiency virus type 1 reverse transcriptase promotes strand transfer J Biol Chem 2006 281 32113 32121 10.1074/jbc.M604665200 16926150

